# Full-fat insect meals in ruminant nutrition: in vitro rumen fermentation characteristics and lipid biohydrogenation

**DOI:** 10.1186/s40104-022-00792-2

**Published:** 2022-12-20

**Authors:** Manuela Renna, Mauro Coppa, Carola Lussiana, Aline Le Morvan, Laura Gasco, Gaelle Maxin

**Affiliations:** 1 Department of Veterinary Sciences, University of Turin, Largo P. Braccini 2, 10095 Grugliasco, TO Italy; 2 Independent Researcher, Université Clermont Auvergne, INRAE, VetAgro Sup, UMR 1213 Herbivores, Saint-Genès-Champanelle, France; 3 Department of Agricultural, Forest and Food Sciences, University of Turin, Largo P. Braccini 2, 10095 Grugliasco, TO Italy; 4 Université Clermont Auvergne, INRAE, VetAgro Sup, UMR 1213 Herbivores, Saint-Genès-Champanelle, France

**Keywords:** Ammonia, Fatty acids, In vitro digestibility, Methane, Nutritive value

## Abstract

**Background:**

The most used protein sources in ruminant nutrition are considered as having negative impacts in terms of environmental sustainability and competition with human nutrition. Therefore, the investigation of alternative and sustainable feedstuffs is becoming a priority in ruminant production systems.

**Results:**

This trial was designed to evaluate eight full-fat insect meals (*Acheta domesticus* – ACD; *Alphitobius diaperinus* – ALD; *Blatta lateralis* – BL; *Gryllus bimaculatus* – GB; *Grylloides sygillatus* – GS; *Hermetia illucens* – HI; *Musca domestica* – MD; and *Tenebrio molitor* – TM) as potential protein and lipid sources in ruminant nutrition. Fermentation parameters and fatty acids (FA) of rumen digesta after 24-h in vitro ruminal incubation of the tested insect meals were measured and compared with those of three plant-based meals (soybean meal, rapeseed meal and sunflower meal) and fishmeal (FM). Similarly to FM, the insect meals led to a significantly lower total gas production (on average, 1.75 vs. 4.64 mmol/g dry matter—DM), methane production (on average, 0.33 vs. 0.91 mmol/g DM), volatile FA production (on average, 4.12 vs. 7.53 mmol/g DM), and in vitro organic matter disappearance (on average, 0.32 vs. 0.59 g/g) than those observed for the plant meals. The insect meals also led to lower ammonia of rumen fluid, when expressed as a proportion of total N (on average, 0.74 vs. 0.52 for the plant and insect meals, respectively), which could be an advantage provided that intestinal digestibility is high. Differences in ruminal fermentation parameters between the insect meals could be partially explained by their chitin, crude protein and ether extract contents, as well as by their FA profile. In particular, high content of polyunsaturated FA, or C12:0 (in HI), seems to partially inhibit the ruminal fermentations.

**Conclusions:**

The tested full-fat insect meals appear to be potentially an interesting protein and lipid source for ruminants, alternative to the less sustainable and commonly used ones of plant origin. The FA profile of the rumen digesta of ACD, ALD, GB, GS and TM, being rich in n-6 polyunsaturated FA, could be interesting to improve the quality of ruminant-derived food products.

**Supplementary Information:**

The online version contains supplementary material available at 10.1186/s40104-022-00792-2.

## Background

Soybean meal (SBM) represents the most used protein source in livestock production. However, it is considered as having a huge impact in terms of environmental sustainability [1] and competition with human nutrition [2]. Even if lower negative impacts are attributed to other protein sources of vegetal origin, such as the rapeseed meal [3], the investigation of alternative and sustainable feeds has become a priority in ruminant production systems [4].

Insects are considered as very promising innovative feedstuffs [5]. Advantages are related to their valuable chemical composition: being rich primarily in proteins and secondly in lipids, they can be used as protein and energy source in diet formulations [6]. Moreover, insects are also considered as more sustainable, when compared to other commodities, as they can be reared on substrates non otherwise valorised, such as organic waste [7], reducing the environmental impact of feed production.

The use of insects for monogastric feeding is increasing worldwide [5]. Even if their use for ruminant nutrition is limited in some countries due to the potential risk of Transmissible Spongiform Encephalopathies for humans [8], the interest in their use is growing up, as they are seen as promising alternative protein sources [9].

Literature on insect meals has been mainly devoted to monogastric species. Generally speaking, the insect meals used were able to sustain growth and performance parameters of fish, crustaceans [10] and poultry species [11]. Similar findings were observed in pigs, when partially or totally substituting fishmeal (FM) or SBM in the diet [12, 13]. In all farmed species, several publications reported positive health impacts of the dietary inclusion of insect meals due to bioactive compounds able to boost the immune system or to modulate the animal microbiota towards beneficial bacteria [14–16].

Considering that the expected population increase will be predominant in emerging countries [17], where the use of processed animal proteins for ruminant feeds is allowed, research on this topic is of outmost relevance. Research on the use of insects for ruminant feeds is still at an infant stage, with only few articles that have been published to date. Original research primarily focused on the assessment of in vitro fermentation parameters (e.g., [9, 18, 19]). Only two in vivo studies have been performed so far, evaluating the performance and physiological status of goat kids fed cricket meal [20] and the suitability of black soldier fly larvae as protein supplement to beef steers consuming low-quality forages [21]. In these studies, only SBM or soybean oil were used as reference feedstuff and the assessment regarded a limited number of insect species in each trial. Moreover, very limited information is available concerning lipid ruminal biohydrogenation, useful to give supplementary information for the comprehension of ruminal fermentation dynamics, as only insect oils from few species were tested so far [22].

Considering the above-mentioned gaps, the aims of this trial were to investigate the in vitro rumen fermentation characteristics and lipid biohydrogenation of eight full-fat insect meals (some of them being never tested before for ruminant or even monogastric animals), as potential protein sources for ruminant nutrition, comparing them to three different plant-based meals commonly used as protein source in ruminant nutrition and with FM as animal-based reference protein source.

## Methods

The experimental procedures were conducted in accordance with the European Union Directive 2010/63/EU, reviewed by the French local ethics committee (C2E2A, “Comité d’Ethique pour l’Expérimentation Animale en Auvergne”) and authorised by the French Ministry for Research (no. 7138-2016092709177605v6).

### Insect and control meals

Eight full-fat insect meals, deriving from different insect species (*Acheta domesticus* L. – ACD; *Alphitobius diaperinus* Panzer *–* ALD; *Blatta lateralis* Walker – BL; *Grylloides sigillatus* Walker – GS; *Gryllus bimaculatus* De Geer – GB; *Hermetia illucens* L. *–* HI; *Musca domestica* L. – MD; and *Tenebrio molitor* L. *–* TM) were used in this study. Due to differences in the life cycle of the different orders of the Insecta class, the ALD, HI, MD and TM meals were obtained from the larval stage, BL meal was obtained from the subadult stage, and the ACD, GB and GS meals were obtained from the adult stage. The insect meals were provided by different suppliers. In details, ACD, GS and GB were provided by Prof. Attawit Kovithvadi (Kasetsart University, Bangkok, Thailand), ALD meal was purchased from Protifarm (Ermelo, The Netherlands), HI meal was obtained from EntoFood (Kuala Lumpur, Malaysia), TM meal was sourced from the National Research Council (Sassari, Italy) after being processed at Porto Conte Ricerche (Sassari, Italy), while BL and MD were raised at the experimental facility of the Department of Agricultural, Forest and Food Sciences (DISAFA) of the University of Turin (Carmagnola (TO), Italy). All the insects were raised on vegetable by-products. No specific indications were provided for HI, TM, ALD, ACD, GB and GS meals regarding the technological processes applied to obtain insect-derived processed animal proteins, as such information is covered by intellectual properties.

Soybean meal, rapeseed meal (RPM) and sunflower meal (SFM) (purchased from F.lli Borello S.r.l., Bra (CN), Italy) were chosen as plant-derived control meals, due to their large use as protein sources in ruminant nutrition [23]. Fishmeal (purchased from NaturAlleva, Cologna Veneta (VR), Italy) was chosen as animal-derived control meal, as it is the reference animal-derived protein source to which insects are usually compared for monogastric animals [6].

### In vitro fermentation

In vitro fermentations were performed using a batch technique, which consists in the incubation of the substrate with rumen fluid and buffer in a vessel placed in water batch. Rumen fluid was collected from four cannulated sheep (Texel, adult castrated males, 64.6 ± 7.10 kg of body weight) housed indoors at the INRAE research farm (INRAE Herbipôle, 63,122 Saint-Genès-Champanelle, France). The sheep were fed permanent grassland hay (840 g dry matter (DM)/head/d) and concentrate (360 g DM/head/d), in two equal meals at 08:30 h and 16:00 h, starting 15 d before the beginning of the trial. The animals had free access to fresh water and minerals. Four independent incubation runs of 24 h (statistical replications) were performed over two consecutive weeks. In each incubation run, all the meal samples (the 8 insect meals and the 4 control meals) were incubated using the rumen fluid from one sheep. A pre-test enabled us to verify that a 24-h incubation time can discriminate the meals and allow interpreting reliable results. On each day of incubation, a sample of the solid fraction of the rumen content from the sheep was collected from the rumen cannula immediately before the morning meal, transferred in less than 10 min to the laboratory into a Thermos flask and then squeezed through a layer of nylon cloth to obtain the rumen fluid to be used as an inoculum in the in vitro incubations. Rumen fluid was diluted 1:2 (v/v) in anaerobic phosphate:carbonate buffer solution prepared as described by Goering and Van Soest [24] modified by Mould et al. [25] and Niderkorn et al. [26] with the following modifications: no NH_4_HCO_3_ was used in the preparation of the bicarbonate buffer, which consisted entirely of NaHCO_3_; MgSO_4_ was substituted by MgCl_2_. This formula enabled to optimize use of N from substrate by rumen microbes. For each incubation, a subsample of 600 ± 0.5 mg DM of each meal sample was individually placed in a 120-mL serum bottle (two bottles per meal sample; i.e., technical replication), pre-warmed at 39 °C and flushed with N_2_. A total volume of 40 mL of buffered rumen fluid was then added. The bottle was sealed hermetically with a butyl rubber stopper and an aluminium crimp seal and immediately manually shaken. All the bottles were incubated in a water bath at 39 °C for 24 h. Blank bottles containing only buffered rumen fluid were incubated simultaneously. Bottles were manually shaken 1 h 30 min, 3 h, 5 h, 7 h and 23 h 30 min after the beginning of the fermentation. Gas production in each bottle was recorded after 3 h and 24 h by using the pressure transducer technique [27]. A gas sample was also taken from the headspace of each serum bottle at 24 h, by using a syringe equipped with a two ways-stopcock. Gas composition was measured immediately after sampling. Fermentation was stopped after 24 h and the bottle contents were transferred to a pre-weighed 50 mL flat-cap conical polypropylene centrifuge tube. The pH was immediately measured, and the tube was centrifuged at 3000 × *g* for 10 min at 4 °C. An aliquot of the supernatant (1.6 mL) was transferred to a polypropylene tube containing 0.16 mL of H_3_PO_4_ 5% (v/v) and frozen at –20 °C for ammonia analysis. A second aliquot of supernatant (0.8 mL) was transferred to a microtube containing 0.5 mL of deproteinising solution (crotonic acid 0.4% w/v, metaphosphoric acid 2% w/v, in HCl 0.5 mol/L). The mixture was cooled at 4 °C for 2 h, centrifuged at 16,500 × *g* for 10 min at 4 °C, and the resulting supernatant was frozen at –20 °C for volatile fatty acid (VFA) analysis. The pellet containing non-degraded particles resulting from the first centrifugation was washed two times with 10 mL of distilled water, centrifuged at 3000 × *g* for 10 min at 4 °C to eliminate the supernatant, and then dried at 60 °C for 48 h.

Four additional incubation runs were performed to determine the fatty acid (FA) composition of the non-degraded particles residues. These incubation runs were carried out following the previously described procedure. The residues were freeze-dried and ground in a micro hammer mill through a 1-mm sieve for FA determination.

### Laboratory analysis

#### Chemical composition of insect and control meals

The insect and control meals were ground using a knife mill (Grindomix GM200, Retsch GmbH, Haan, Germany; final fineness < 300 μm). AOAC International [28] procedures were used to determine DM (method no. 930.15), ash (method no. 942.05), crude protein (CP, method no. 984.13), acid detergent fibre and acid detergent lignin (ADF and ADL, method no. 973.18). The CP content of the insect meals was calculated using the nitrogen to protein (N:P) conversion factor of 4.67 for HI meal, 4.75 for TM meal, 4.86 for ALD and 4.76 for MD [29]. Following the findings of Ritvanen et al. [30], the N:P conversion factors of 5.09 and 5.00 were used for ACD meal and GB meal, respectively. As no specific N:P conversion factor can be found in literature for GS and BL, we decided to use the value 5.00 for both meals, considering that (i) similarly to ACD and GB, GS belongs to the order Orthoptera, family Gryllidae, subfamily Gryllinae, and (ii) BL does not present a larval stage. The CP content of the control meals was calculated using the conventional N:P conversion factor of 6.25. Ether extract (EE, method no. 2003.05) was analysed according to AOAC International [31]. Neutral detergent fibre (NDF) was analysed according to Mertens [32]; α-amylase (Merck, Darmstadt, Germany) and sodium sulphite (Merck, Darmstadt, Germany) were added, and results were corrected for residual ash content. Neutral-detergent insoluble nitrogen (NDIN) and acid-detergent insoluble nitrogen (ADIN) were determined according to Licitra et al. [33].

Chitin was estimated according to Finke [34] by correction considering the amino acid content of the ADF fraction and assuming the remainder of the ADF fraction is chitin. The proximate composition of the insect and control meals was expressed as g/kg DM.

The FA composition of the insect and control meals was assessed as reported in Renna et al. [35]. A combined direct transesterification and solid-phase extraction method was used [36]. Fatty acid methyl esters were separated and quantified by a high-resolution gas chromatograph (Shimadzu GC 2010 Plus; Shimadzu, Kyoto, Japan) equipped with a flame-ionisation detector, and a CP-Sil 88 capillary column (100 m × 0.25 mm ID, 0.20 μm film thickness; Varian Inc., Palo Alto, CA, USA). Injections were made in on-column mode and the injection volume was 0.5 μL. The temperatures of the injector and the flame-ionisation detector were maintained at 250 and 280 °C, respectively. The column temperature was held at 45 °C for 5 min, then raised 20 °C/min up to 195 °C and maintained for 65 min. Peaks identification and quantification were performed as reported by Renna et al. [37]. The results were expressed as g/100 g total FA, except for total FA which were expressed as g/kg DM.

#### Chemical composition of rumen content

Volatile fatty acids were determined by gas chromatography (Perkin Elmer Clarus 580 GC, Perkin Elmer, Waltham, MA, USA, equipped with an Agilent column CP-WAX 58 FFAP 25 m × 0.25 mm, Agilent, Santa Clara, CA, USA) according to Jouany [38]. Ammonia was measured using the Berthelot reaction [39]. The composition of fermentation gases (CH_4_, CO_2_ and H_2_) was determined by gas chromatography (MicroGC Fusion, Inficon, East Syracuse, NY, USA) as detailed in Macheboeuf et al. [40]. The residue containing non-degraded particles was oven-dried at 60 °C for 48 h, weighed and then incinerated at 550 °C to determine the organic matter content. This procedure allowed the calculation of in vitro organic matter disappearance (IVOMD).

The FA composition of the rumen content was determined as detailed in Alves et al. [41]. The samples were then analysed using a high-resolution gas chromatograph (Shimadzu GC 2010 Plus; Shimadzu, Kyoto, Japan) equipped with a flame-ionisation detector and a CP-Sil 88 capillary column (100 m × 0.25 mm ID, 0.20 μm film thickness; Varian Inc., Palo Alto, CA, USA). The temperatures of the injector and the flame-ionisation detector were maintained at 250 and 280 °C, respectively. The column temperature was held at 100 °C for 1 min, then raised 50 °C/min up to 150 °C and maintained for 20 min, then raised 1 °C/min up to 190 °C for 5 min and, at the end, raised 1 °C/min up to 200 °C for 35 min. Peaks were identified by comparing the retention times to pure standards (Restek Corporation, Bellefonte, PA, USA; Supelco Inc., Bellefonte, PA, USA; Matreya LLC, Pleasant Gap, PA, USA) and by comparison with published chromatograms [41]. The results were expressed as g/100 g total FA.

### Calculations and statistical analyses

For the in vitro fermentations, the total number of observations was 96 (12 meals × 4 incubation runs (statistical replications) × 2 technical replications (two bottles per sample and per incubation run). The results of the two technical repetitions were averaged for statistical analyses.

By subtracting the results from blank cultures (ruminal buffered fluid without meal samples), the amount of feed degraded and the net production of fermentation end-products (VFA and ammonia) at the end of each incubation period were calculated.

All data from the in vitro fermentation and rumen content were analysed by a GLM ANOVA procedure in SPSS (version 27.0 for Windows; SPSS Inc., Chicago, IL, USA). Data normality was verified using the Shapiro–Wilk test, while homogeneity of variances was visually verified with graphics of the residuals. The model included meal type (12 levels) as fixed effect and incubation run (4 levels) as random effect. Least squares means were reported with the pooled standard error of the mean derived from the model. Differences among the means were adjusted for multiple comparisons using Tukey–Kramer’s method and were declared significant at *P*-values < 0.05. 0.05 < *P* values ≤ 0.10 were interpreted as a trend toward significance.

No statistical analysis was conducted to compare the chemical composition of the tested meals as each meal derived from a single commercial batch.

## Results

### Chemical composition and fatty acid profile of the meals

All the insect meals contained high CP content (Table 1), which ranged from 339 g/kg DM for TM to 505 g/kg DM for GS; HI, BL and MD had a CP content ranging from 351 to 408 g/kg DM, while in ALD, GB and ACD the CP content ranged from 457 to 496 g/kg DM. Regarding the control meals, the CP content ranged from 324 to 748 g/kg DM in SFM and FM, respectively. The insect meals had a high EE content (above 200 g/kg DM) when compared to the other meals (lower than 100 g/kg DM). The fibre contents evaluated with NDF concentrations were high for SFM and RPM (equal than or above 306 g/kg DM). The insect meals had NDF contents ranging from 225 to 282 g/kg DM, while SBM showed about half NDF content than the insect meals. Fishmeal did not contain fibre. The average estimated chitin content in the insect meals was 61 g/kg DM, with values ranging from 51 g/kg DM for TM to 78 g/kg DM for BL.

**Table 1:** Chemical composition (g/kg DM, unless otherwise stated) of the insect and control meals

Item	**Meals**											
	**Full-fat insect meals**								**Control meals**			
	**ACD**	**ALD**	**BL**	**GB**	**GS**	**HI**	**MD**	**TM**	**SBM**	**RPM**	**SFM**	**FM**
DM, g/kg	934	941	930	912	927	953	937	940	881	903	894	915
OM	939	951	961	951	951	911	931	961	922	928	922	848
CP	496	457	402	483	505	351	408	339	559	407	324	748
EE	207	245	319	203	205	269	238	392	6	28	17	99
NDF	254	246	250	260	260	282	280	225	113	306	381	0
ADF	84	58	83	93	77	79	70	82	68	199	273	0
ADL	18	12	20	8	7	15	13	20	2	70	79	0
NDIN	36	24	35	40	30	31	29	35	24	44	46	0
ADIN	33	21	31	37	23	26	24	32	18	21	31	0
Chitin	67	54	78	59	63	52	61	51	n.a.	n.a.	n.a.	n.a.

Abbreviations: *DM* Dry matter, *ACD Acheta domesticus*, *ALD Alphitobius diaperinus*, *BL Blatta lateralis*, *GB Gryllus bimaculatus*, *GS Grylloides sigillatus*, *HI Hermetia illucens*, *MD Musca domestica*, *TM Tenebrio molitor*, *SBM* Soybean meal, *RPM* Rapeseed meal, *SFM* Sunflower meal, *FM* Fishmeal, *OM* Organic matter, *CP* Crude protein, *EE* Ether extract, *NDF* Neutral detergent fibre, *ADF* Acid detergent fibre, *ADL* Acid detergent lignin, *NDIN* Neutral detergent insoluble nitrogen, *ADIN* Acid detergent insoluble nitrogen, *n.a*. not analysed

The total FA content (Table 2) in the insect meals was higher than 144 g/kg DM, with 317 g/kg DM in TM, whereas in the control meals it ranged from 6 g/kg DM in SBM to 68 g/kg DM in FM. The proportion of saturated fatty acids (SFA) in the insect meals (except for TM) and FM was equal or higher than 29 g/100 g FA, with BL and HI having values higher than 50 g/100 g FA, whereas SFA proportion was equal or lower than 27 g/100 g FA in the plant meals and in TM. The HI meal showed high proportions of C12:0 and C14:0, and the BL showed high proportions of C16:0 and C18:0. Both GB and TM contained mainly C18:1 *c*9. The main FA in ACD, ALD, GS and MD were C16:0, C18:1 *c*9 and C18:2 n-6. The insect meals contained very low proportions of n-3 polyunsaturated fatty acids (PUFA). Rapeseed meal and SFM were mainly composed by C18:1 *c*9 and C18:2 n-6, whereas the main FA in SBM were C16:0 and C18:2 n-6. The main FA in FM was C16:0 and it was rich in long-chain n-3 FA, their sum reaching about one third of total detected FA.

**Table 2:** Fatty acid composition of the insect and control meals (g/100 g FA, unless otherwise stated)

Item	**Meals**											
	**Full-fat insect meals**								**Control meals**			
	**ACD**	**ALD**	**BL**	**GB**	**GS**	**HI**	**MD**	**TM**	**SBM**	**RPM**	**SFM**	**FM**
C10:0	0.34	0.42	0.44	0.17	0.34	0.26	0.33	0.22	2.79	1.88	2.63	0.79
C12:0	0.07	0.03	0.11	0.18	0.13	26.8	0.10	0.30	0.03	0.02	0.02	0.10
C14:0	0.53	0.78	0.99	1.43	0.71	6.08	2.32	3.10	0.19	0.18	0.10	6.75
C16:0	25.4	29.3	31.8	21.5	25.7	20.0	22.4	17.2	18.6	8.69	8.05	22.5
C16:1 *c*9	0.70	0.32	1.01	1.72	1.45	1.70	11.1	1.63	0.15	1.32	0.13	8.06
C18:0	9.28	9.11	15.8	5.11	7.89	2.97	4.46	2.90	5.09	1.73	3.46	6.64
C18:1 c9	25.5	35.6	24.3	48.6	27.6	10.8	25.8	43.2	11.3	42.5	53.7	10.2
C18:2 n-6	34.6	20.5	21.6	15.4	32.0	24.5	19.3	28.0	51.9	25.4	28.0	1.47
C18:3 n-3	0.90	0.76	0.43	2.97	0.77	2.01	1.90	1.42	7.19	4.45	1.48	0.58
C18:3 n-6	0	0.01	0.01	0.03	0.04	0.01	0.07	0.01	0.06	0.06	0	0.21
C20:0	0.56	0.56	1.02	0.28	0.81	0.12	0.20	0.19	0.27	0.44	0.45	0.33
C22:0	0.13	0.25	0.21	0.11	0.14	0.24	0.14	0.04	0.35	0.27	0.69	0.14
C20:5 n-3	0.12	0	0.24	0.05	0.08	0	0.33	0	0	0	0	14.4
C22:5 n-3	0	0	0	0	0	0	0	0	0	0	0	2.06
C22:6 n-3	0	0	0	0	0	0	0	0	0	0	0	13.9
Other^1^	1.86	2.38	2.05	2.51	2.29	4.58	11.55	1.79	2.10	13.0	1.32	11.9
Total SFA	36.4	40.7	50.6	29.0	35.9	56.8	31.1	24.1	27.4	13.4	15.5	37.9
Total BCFA^2^	0.57	0.81	0.65	0.66	0.89	0.90	5.72	0.78	0	0	0	1.27
Total MUFA	27.0	37.3	26.5	51.4	30.2	14.0	40.7	45.7	13.4	56.6	55.0	24.8
Total PUFA	36.0	21.3	22.3	18.9	33.0	28.3	22.5	29.5	59.2	30.0	29.5	36.1
Total FA, g/kg DM	168	155	144	203	163	209	194	317	6	27	17	68

Abbreviations: *FA* Fatty acids, *ACD Acheta domesticus*, *ALD Alphitobius diaperinus*, *BL Blatta lateralis*, *GB Gryllus bimaculatus*, *GS Grylloides sigillatus*, *HI Hermetia illucens*, *MD Musca domestica*, *TM Tenebrio molitor*, *SBM* Soybean meal, *RPM* Rapeseed meal, *SFM* Sunflower meal, *FM* Fishmeal, *c*: *cis*, *SFA* Saturated fatty acids, *BCFA* Branched-chain fatty acids, *MUFA* Monounsaturated fatty acids, *PUFA* Polyunsaturated fatty acids, *DM* Dry matter

^1^Other: C14:1 *c*9 + C15:0 + C15 *iso* + C15 *aiso* + C16 *iso* + C17:1 *c*9 + C17 *iso* + C17 *aiso* + ∑C18:1 *t* + C18:1 *c*11 + C18:1 *c*12 + C18:1 *c*14 + C18:1 *t*16 + C20:1 *c*9 + C20:1 *c*11 + C20:2 n-6 + C20:3 n-6 + C22:1 n-9 + C20:3 n-3 + C20:4 n-6

^2^Total BCFA: C15 *iso* + C15 *aiso* + C16 *iso* + C17 *iso* + C17 *aiso*

### In vitro rumen fermentation parameters

The fermentation characteristics obtained after 24 h of fermentation varied with the meals for all the evaluated parameters (Table 3).

**Table 3:** In vitro rumen fermentation characteristics after 24 h of incubation of the insect and control meals

Item	**Meals**													
	**Full-fat insect meals**								**Control meals**					
	**ACD**	**ALD**	**BL**	**GB**	**GS**	**HI**	**MD**	**TM**	**SBM**	**RPM**	**SFM**	**FM**	**SEM**	***P-*value**
Total gas 24 h, mmol/g DM	1.50^e^	2.02^d^	2.42^d^	1.48^g^	1.64^fg^	1.55^fg^	1.68^f^	1.70^f^	5.41^a^	5.03^b^	3.47^c^	1.10^h^	0.051	0.001
Methane 24 h														
mmol/g DM	0.262^fg^	0.408^e^	0.522^d^	0.243^fg^	0.316^f^	0.260^fg^	0.290^fg^	0.306^f^	1.158^a^	0.933^b^	0.647^c^	0.185^g^	0.018	< 0.001
mmol/mmol total gas^1^	0.174^def^	0.200^ab^	0.215^a^	0.164^f^	0.192^bc^	0.167^f^	0.172^def^	0.181^cdef^	0.214^a^	0.186^bcd^	0.187^bcd^	0.168^ef^	0.039	< 0.001
Final pH	7.0^a^	7.0^a^	7.0^a^	7.0^a^	7.0^a^	7.0^a^	7.0^a^	7.0^a^	6.8^bc^	6.6^d^	6.7^c^	7.1^a^	0.04	< 0.001
Total VFA, mmol/g DM	3.92^fg^	4.70^e^	5.37^d^	3.70^g^	4.15^f^	3.57^g^	3.91^fg^	3.66^g^	8.80^a^	7.82^b^	5.98^c^	3.89^fg^	0.08	< 0.001
Acetate, g/100 g VFA	57.3^bc^	56.3^cd^	53.5^e^	56.1^cd^	58.0^bc^	56.9^cd^	56.3^cd^	55.4^d^	57.0^cd^	59.0^b^	61.3^a^	53.4^e^	0.46	< 0.001
Propionate, g/100 g VFA	17.8^de^	16.6^ef^	16.5^ef^	20.1^b^	16.0^f^	19.1^bcd^	19.7^bc^	20.4^b^	22.6^a^	23.3^a^	22.2^a^	18.4^cd^	0.34	< 0.001
Butyrate, g/100 g VFA	9.2^cd^	9.5^cd^	10.5^b^	8.7^d^	9.1^cd^	12.1^a^	10.9^b^	9.1^cd^	9.7^c^	8.9^cd^	6.9^e^	10.8^b^	0.021	< 0.001
Acetate:propionate ratio	3.22^bc^	3.40^ab^	3.26^b^	2.80^de^	3.62^a^	2.98^cd^	2.87^d^	2.72^de^	2.53^e^	2.54^e^	2.76^d^	2.91^d^	0.06	< 0.001
IVOMD, g/g	0.28^f^	0.35^de^	0.50^c^	0.25^g^	0.30^ef^	0.24^g^	0.33^de^	0.29^f^	0.70^a^	0.61^b^	0.46^c^	0.32^de^	0.007	< 0.001
NH_3_-N, mmol/g DM	3.55^d^	4.04^c^	4.96^a^	3.08^e^	3.80^cd^	2.49^g^	2.97^ef^	2.75^fg^	4.48^b^	3.06^e^	2.91^ef^	3.59^d^	0.069	< 0.001
NH_3_-N:total-N, mg/mg total N	0.473^efg^	0.580^d^	0.771^b^	0.419^g^	0.494^e^	0.443^eg^	0.480^ef^	0.491^e^	0.702^c^	0.665^cd^	0.866^a^	0.427^f^	0.013	< 0.001

Different superscripts within a row indicate significant differences among the tested meals (*P*-value < 0.05)

Abbreviations: *ACD Acheta domesticus*, *ALD Alphitobius diaperinus*, *BL Blatta lateralis*, *GB Gryllus bimaculatus*, *GS Grylloides sigillatus*, *HI Hermetia illucens*, *MD Musca domestica*, *TM Tenebrio molitor*, *SBM* soybean meal, *RPM* Rapeseed meal, *SFM* Sunflower meal, *FM* Fishmeal, *SEM* Standard error of the mean, *DM* Dry matter, *VFA* Volatile fatty acids, *IVOMD* In vitro organic matter disappearance

^1^This characteristic corresponds to the proportion of methane in the total gas, expressed as mmol of methane produced in the total mmol of gas produced

The total gas production after 24 h of incubation was significantly higher for the plant meals than for the insect meals (on average + 2.89 mmol/g DM). The lowest total gas production value was obtained for FM (–0.65 mmol/g DM on average, compared to insect meals).

The quantity of methane produced after 24 h was significantly higher for the plant meals than for both the insect meals and FM (on average, + 0.65 mmol/g DM). The BL and ALD showed significantly higher values of methane than those obtained for the other insect meals (+ 0.19 mmol/g DM), with BL showing the absolute highest value. When expressed as mmol per mmol of total gas, methane was the highest for SBM and BL (in both cases equal to 0.21 and not significantly different from ALD) and the lowest for GB and HI (0.16), the other meals showing intermediate values.

The insect meals and FM led to a significantly higher final rumen pH than that observed for the plant meals (on average 7.0 vs. 6.7, respectively).

The plant meals showed significantly higher total VFA concentration at the end of the incubation period than the other meals (on average + 3.40 mmol/g DM). The BL and ALD showed higher total VFA values than the other tested insect meals (on average + 1.55 and + 0.88 mmol/g DM, respectively), with BL even showing a higher value when compared to ALD. The proportion of acetate on total VFA was the greatest for SFM (61.3 g/100 g VFA) and the lowest for FM (53.4 g/100 g VFA); the insect meals showed values ranging from 53.5 to 58.0 g/100 g VFA in BL and GS, respectively. The fermentation of plant meals resulted in a higher propionate proportion than the other meals (on average higher than 22.0 g/100 g VFA vs. lower than 18.3 g/100 g VFA). Among the insect meals, the fermentation of GB, TM, MD and HI resulted in higher propionate proportion when compared to ALD, BL and GS (on average + 3.5 g/100 g VFA). The proportion of butyrate was the greatest for HI (12.1 g/100 g VFA) and the lowest for SFM (6.9 g/100 g VFA).

The fermentation of plant meals resulted in a higher IVOMD when compared to FM (on average + 0.27 g/g) or insect meals (on average + 0.30 g/g), except for BL which showed comparable value to that of SFM.

The total ammonia production was the greatest for BL, followed by SBM (4.96 and 4.48 mmol/g DM, respectively). The incubation of HI, TM, MD and SFM resulted in less than 3 mmol/g DM of total ammonia. When ammonia was expressed as a proportion of total N, it was the highest for SFM (0.87), followed by BL (0.77) and SBM (0.70). Intermediate values were observed for RPM (0.66) and ALD (0.58), whereas all the other meals showed values lower than 0.50 mg/mg total N.

### Fatty acid profile of rumen digesta

The results for the FA of rumen digesta after 24 h of incubation for each of the meal tested in this study are reported in Table 4, and the coelution or separation of C18:1 *t*9, *t*10 and *t*11 isomers is given in Additional File 1.

**Table 4:** Fatty acids (g/100 g FA, unless otherwise stated) of ruminal digesta after 24 h incubation of the insect and control meals

Item	Meals												SEM	*P*-value
	Full-fat insect meals								Control meals					
	ACD	ALD	BL	GB	GS	HI	MD	TM	SBM	RPM	SFM	FM		
C10:0	0.10^d^	0.07^d^	0.07^d^	0.06^d^	0.05^d^	0.77^a^	0.05^d^	0.03^d^	0.57^b^	0.20^cd^	0.31^c^	0.28^c^	0.040	< 0.001
C11:0 + C10:1 *c*9	0.03^f^	0.03^f^	0.04^ef^	0.05^ef^	0.05^def^	0.09^de^	0.04^ef^	0.06^def^	0.26^a^	0.17^c^	0.21^b^	0.10^d^	0.012	< 0.001
C12:0	0.42^b^	0.20^b^	0.42^b^	0.38^b^	0.34^b^	19.98^a^	0.35^b^	0.58^b^	1.47^b^	1.26^b^	1.23^b^	0.76^b^	0.846	< 0.001
C13:0	0.005^ab^	0.003^ab^	0.001^b^	0.004^ab^	0.005^ab^	0.009^ab^	0.004^ab^	0.007^ab^	0.021^ab^	0.024^a^	0.012^ab^	0.018^ab^	0.0050	0.021
C14:0	1.65^ef^	1.39^f^	2.43^e^	1.51^f^	2.06^ef^	6.83^b^	3.74^d^	4.05^cd^	4.40^cd^	4.55^c^	4.48^cd^	9.18^a^	0.198	< 0.001
C15:0	0.90^e^	0.68^e^	0.63^e^	0.76^e^	0.68^e^	1.30^d^	1.71^c^	0.70^e^	4.70^a^	4.14^b^	4.82^a^	1.73^c^	0.093	< 0.001
C16:0	31.02^cde^	35.79^ab^	30.2^de^	32.21^cd^	37.64^a^	26.96^e^	32.23^cd^	22.59^f^	31.47^cd^	29.66^de^	27.59^e^	34.00^bc^	0.822	< 0.001
C17:0	0.31^d^	0.62^ab^	0.35^cd^	0.40^bcd^	0.33^cd^	0.48^bcd^	0.58^abc^	0.27^d^	0.75^a^	0.76^a^	0.64^ab^	0.76^a^	0.056	< 0.001
C18:0	13.52^a^	11.29^ab^	12.19^a^	12.91^a^	14.7^a^	7.21^b^	11.26^ab^	7.70^b^	11.35^ab^	10.84^ab^	11.35^ab^	10.96^ab^	0.919	< 0.001
C20:0	0.146^bcd^	0.179^bcd^	0.161^bcd^	0.249^ab^	0.222^abc^	0.111^cd^	0.107^d^	0.087^d^	0.224^abc^	0.324^a^	0.255^ab^	0.255^ab^	0.0240	< 0.001
C21:0 (+ CLA *t*9*c*11)	0.010^cd^	0.030^a^	0.023^ab^	0.034^a^	0.032^a^	0.025^ab^	0.023^abc^	0.016^bc^	0.000^d^	0.000^d^	0.000^d^	0.000^d^	0.0030	< 0.001
C22:0	0.024^d^	0.026^cd^	0.039^bcd^	0.027^cd^	0.024^d^	0.028^cd^	0.044^bcd^	0.034^bcd^	0.125^a^	0.094^ab^	0.090^abc^	0.069^abcd^	0.0130	< 0.001
C24:0	0.017^b^	0.022^b^	0.003^c^	0.020^b^	0.047^a^	0.002^c^	0.014^b^	0.004^c^	0.000^c^	0.000^c^	0.000^c^	0.000^c^	0.0024	< 0.001
C13 *iso*	0.154^b^	0.090^b^	0.094^b^	0.143^b^	0.119^b^	0.168^b^	0.153^b^	0.107^b^	0.799^a^	0.731^a^	0.769^a^	0.243^b^	0.0370	< 0.001
C13 *aiso*	0.027^c^	0.016^c^	0.013^c^	0.018^c^	0.019^c^	0.079^b^	0.022^c^	0.013^c^	0.159^a^	0.127^a^	0.141^a^	0.04^c^	0.100	< 0.001
C14 *iso*	0.67^ cd^	0.36^d^	0.42^d^	0.55^d^	0.54^d^	0.90^bc^	0.49^d^	0.43^d^	2.79^a^	2.70^a^	2.59^a^	1.16^b^	0.077	< 0.001
C15 *iso*	1.41^cd^	0.55^e^	0.53^e^	1.09^cde^	0.96^de^	1.66^c^	0.86^de^	0.74^e^	5.58^a^	4.17^b^	5.26^a^	1.42^cd^	0.143	< 0.001
C15 *anteiso*	2.15^c^	0.90^d^	0.71^d^	1.52^cd^	1.51^cd^	2.26^c^	1.19^cd^	1.31^cd^	8.60^a^	7.42^b^	8.28^ab^	2.18^c^	0.264	< 0.001
C16 *iso*	0.76^cde^	0.35^f^	0.33^f^	0.59^cdef^	0.59^cdef^	0.86^c^	0.50^def^	0.48^ef^	2.18^ab^	2.07^b^	2.45^a^	0.83^cd^	0.076	< 0.001
C17 *iso*	0.25^ef^	0.20^f^	0.19^f^	0.38^e^	0.21^f^	0.36^e^	0.74^cd^	0.16^f^	1.00^a^	0.82^bc^	0.91^ab^	0.63^d^	0.032	< 0.001
C17 *anteiso*	0.68^c^	0.42^d^	0.41^d^	0.53^c^	0.57^c^	0.86^c^	0.44^c^	0.47^c^	2.49^a^	2.25^a^	2.61^a^	1.28^b^	0.094	< 0.001
C18 *iso*	0.06^e^	0.35^b^	0.06^e^	0.03^e^	0.04^e^	0.17^cd^	0.58^a^	0.25^c^	0.03^e^	0.05^e^	0.02^e^	0.09^de^	0.023	< 0.001
C12:1 *c*9	0.09^d^	0.06^d^	0.05^d^	0.08^d^	0.09^d^	0.14^cd^	0.12^cd^	0.11^d^	0.63^a^	0.45^b^	0.47^b^	0.21^c^	0.200	< 0.001
C14:1 *t*9	0.09^c^	0.08^c^	0.11^c^	0.08^c^	0.15^c^	0.05^c^	0.32^a^	0.24^b^	0.12^c^	0.08^c^	0.05^c^	0.08^c^	0.022	< 0.001
C16:1 *c*7	0.6^bcd^	0.77^bcd^	0.43^d^	0.52^cd^	0.58^bcd^	0.58^bcd^	2.96^a^	1.07^bcd^	1.18^bc^	1.20^b^	1.14^b^	0.91^bcd^	0.136	< 0.001
C16:1 *c*9	0.47^b^	0.25^b^	1.22^b^	1.10^b^	0.85^b^	1.10^b^	7.01^a^	1.10^b^	0.43^b^	0.55^b^	0.33^b^	6.58^a^	0.224	< 0.001
C17:1 *c*9	0.025^ab^	0.039^ab^	0.028^ab^	0.018^b^	0.024^ab^	0.023^ab^	0.043^a^	0.022^ab^	0.032^ab^	0.020^ab^	0.023^ab^	0.022^ab^	0.0050	0.044
C17:1 *t*10	0.026^f^	0.108^bc^	0.087^cd^	0.045^ef^	0.039^ef^	0.087^cd^	0.149^a^	0.071^de^	0.038^ef^	0.028^f^	0.033^f^	0.124^ab^	0.0080	< 0.001
C18:1 *t*4	0.008^bc^	0.018^abc^	0.020^abc^	0.024^ab^	0.008^bc^	0.012^abc^	0.032^a^	0.009^bc^	0.000^c^	0.000^c^	0.000^c^	0.000^c^	0.0046	< 0.001
C18:1 *t*5	0.015^bc^	0.018^abc^	0.023^ab^	0.029^ab^	0.018^abc^	0.013^bc^	0.033^a^	0.016^abc^	0.000^c^	0.000^c^	0.000^c^	0.000^c^	0.0038	< 0.001
C18:1 *t*6-8	0.130^bc^	0.077^c^	0.272^ab^	0.219^abc^	0.044^c^	0.125^bc^	0.365^a^	0.091^c^	0.072^c^	0.145^bc^	0.097^c^	0.071^c^	0.0350	< 0.001
C18:1 *t*9-11	3.13^abc^	3.57^ab^	4.18^a^	4.52^a^	1.81^bc^	3.14^abc^	4.41^a^	3.30^abc^	1.73^bc^	1.94^bc^	1.82^bc^	1.33^c^	0.442	< 0.001
C18:1 *t*12	0.141^abc^	0.080^bc^	0.120^ab^	0.196^ab^	0.033^c^	0.101^bc^	0.273^a^	0.071^bc^	0.022^c^	0.033^c^	0.021^c^	0.055^bc^	0.0330	< 0.001
C18:1 *t*13 + *t*14	0.28^bc^	0.14^bc^	0.28^bc^	0.39^ab^	0.11^c^	0.24^bc^	0.58^a^	0.12^c^	0.08^c^	0.19^bc^	0.14^c^	0.24^bc^	0.530	< 0.001
C18:1 *c*9 + *c*10 + *t*15	12.37^ef^	21.52^c^	30.09^a^	13.90^de^	15.50^d^	7.98^g^	13.95^de^	27.50^b^	4.46^h^	11.26^f^	12.68^ef^	7.92^g^	0.627	< 0.001
C18:1 *c*11	0.55^c^	0.46^c^	0.48^c^	0.55^c^	0.56^c^	0.66^c^	0.52^c^	0.49^c^	1.11^b^	2.71^a^	0.97^b^	2.67^a^	0.075	< 0.001
C18:1 *c*12	0.085^c^	0.089^c^	0.051^c^	0.073^c^	0.060^c^	0.168^b^	0.202^a^	0.063^c^	0.170^b^	0.173^b^	0.152^b^	0.067^c^	0.0100	< 0.001
C18:1 *c*13	0.049^bc^	0.063^abc^	0.073^abc^	0.089^ab^	0.114^a^	0.055^bc^	0.030^bc^	0.018^c^	0.050^bc^	0.042^bc^	0.038^bc^	0.048^bc^	0.0120	< 0.001
C18:1 *c*14 + *t*16	0.079^b^	0.191^a^	0.142^ab^	0.194^a^	0.032^b^	0.064^b^	0.196^a^	0.041^b^	0.044^b^	0.063^b^	0.054^b^	0.049^b^	0.2300	< 0.001
C20:1 *c*11	0.013^de^	0.063^bc^	0.041^cde^	0.025^cde^	0.023^cde^	0.038^cde^	0.037^cde^	0.026^cde^	0.000^e^	0.093^b^	0.055^bcd^	0.369^a^	0.0104	< 0.001
C18:2 *t*11*t*15	0.068^a^	0.033^abc^	0.060^ab^	0.033^abc^	0.059^ab^	0.026^bc^	0.053^ab^	0.064^ab^	0.000^c^	0.000^c^	0.000^c^	0.000^c^	0.0086	< 0.001
C18:2 *t*9*t*12	0.016^b^	0.013^b^	0.026^b^	0.033^b^	0.011^b^	0.025^b^	0.019^b^	0.026^b^	0.000^b^	0.000^b^	0.000^b^	0.140^a^	0.0101	< 0.001
C18:2 *t*9*c*13 (+ *t*8*c*12)	0.083^bc^	0.299^a^	0.044^cd^	0.078^bcd^	0.136^b^	0.028^cd^	0.024^cd^	0.030^cd^	0.000^d^	0.000^d^	0.000^d^	0.063^cd^	0.0162	< 0.001
C18:2 *c*9*t*12 + c18:1 *c*16	0.64^bcde^	0.72^bcd^	0.20^g^	0.49^defg^	0.61^cde^	0.36^fg^	0.29^g^	0.44^efg^	0.77^bc^	0.91^b^	1.17^a^	0.42^fg^	0.063	< 0.001
C18:2 *t*9*c*12	0.23^bc^	0.57^a^	0.19^c^	0.18^c^	0.27^b^	0.19^c^	0.17^c^	0.06^d^	0.06^d^	0.08^d^	0.03^d^	0.05^d^	0.018	< 0.001
C18:2 *t*11*c*15	0.010	0.016	0.020	0.013	0.012	0.031	0.012	0.013	0.049	0.172	0.032	0.077	0.0390	0.225
C18:2 n-6	22.3^a^	12.76^d^	6.38^ef^	18.02^bc^	15.50^cd^	8.96^e^	7.40^ef^	19.97^ab^	7.76^ef^	5.45^f^	4.89^fg^	1.73^g^	0.852	< 0.001
C18:2 *c*9*c*15	0.037^a^	0.028^ab^	0.027^ab^	0.020^ab^	0.025^ab^	0.016^ab^	0.018^ab^	0.026^ab^	0.000^b^	0.000^b^	0.000^b^	0.023^b^	0.0072	0.009
CLA *c*9*t*11 (+ *t*7*c*9 + *t*8*c*10)	0.055^c^	0.042^c^	0.367^ab^	0.308^ab^	0.049^c^	0.194^bc^	0.180^bc^	0.533^a^	0.000^c^	0.000^c^	0.000^c^	0.000^c^	0.0549	< 0.001
CLA *t*11*c*13 (+ *c*9*c*11)	0.010^bc^	0.007^bc^	0.007^bc^	0.014^ab^	0.010^bc^	0.024^a^	0.015^ab^	0.012^b^	0.000^c^	0.000^c^	0.000^c^	0.000^c^	0.0024	< 0.001
CLA *t*10*c*12	0.011^cd^	0.01^cd^	0.056^a^	0.022^bcd^	0.018^cd^	0.051^a^	0.025^bc^	0.040^ab^	0.000^d^	0.000^d^	0.000^d^	0.000^d^	0.0048	< 0.001
CLA *t*10*t*12 (+ *t*11*t*13)	0.044^d^	0.112^c^	0.131^abc^	0.173^ab^	0.158^abc^	0.184^a^	0.166^abc^	0.114^bc^	0.000^d^	0.000^d^	0.000^d^	0.000^d^	0.0149	< 0.001
C18:3 n-3	0.81^bcd^	0.66^cd^	1.14^b^	0.52^d^	0.54^d^	1.05^bc^	0.71^cd^	0.91^bcd^	2.05^a^	2.01^a^	1.75^a^	0.90^bcd^	0.086	< 0.001
C20:2 n-6	0.018^c^	0.015^c^	0.034^c^	0.026^c^	0.008^c^	0.015^c^	0.014^c^	0.011^c^	0.174^b^	0.079^bc^	0.083^bc^	0.759^a^	0.0280	< 0.001
C20:4 n-6	0.084^c^	0.000^d^	0.124^b^	0.025^d^	0.020^d^	0.030^d^	0.102^bc^	0.003^d^	0.000^d^	0.000^d^	0.000^d^	0.509^a^	0.0100	< 0.001
C20:5 n-3	0.000^b^	0.000^b^	0.000^b^	0.000^b^	0.000^b^	0.000^b^	0.000^b^	0.000^b^	0.000^b^	0.000^b^	0.000^b^	5.11^a^	0.0310	< 0.001
C22:5 n-3	0.000^b^	0.000^b^	0.000^b^	0.000^b^	0.000^b^	0.000^b^	0.000^b^	0.000^b^	0.000^b^	0.000^b^	0.000^b^	0.33^a^	0.0100	< 0.001
C22:6 n-3	0.000^b^	0.000^b^	0.000^b^	0.000^b^	0.000^b^	0.000^b^	0.000^b^	0.000^b^	0.000^b^	0.000^b^	0.000^b^	3.17^a^	0.0600	< 0.001
Total SFA	48.13^ef^	50.29^de^	46.53^f^	48.56^def^	56.15^b^	63.77^a^	50.12^def^	36.12^g^	55.35^bc^	52.01^cd^	50.98^de^	58.12^b^	0.941	< 0.001
Total OCFA	1.27^e^	1.48^de^	1.11^e^	1.25^e^	1.11^e^	1.92^d^	2.50^c^	1.08^e^	5.55^a^	4.98^b^	5.52^a^	2.65^c^	0.122	< 0.001
Total BCFA	6.15^cde^	3.23^f^	2.76^f^	4.84^def^	4.55^ef^	7.32^cd^	4.98^def^	3.96^ef^	23.62^a^	20.32^b^	23.02^a^	7.88^c^	0.555	< 0.001
Total *iso* BCFA	3.30^cd^	1.90^ef^	1.62^f^	2.78^de^	2.45^def^	4.12^c^	3.33^cd^	2.17^ef^	12.38^a^	10.53^b^	11.99^a^	4.37^c^	0.241	< 0.001
Total *anteiso* BCFA	2.85^cde^	1.33^ef^	1.14^f^	2.06^cdef^	2.09^cdef^	3.20^cd^	1.65^def^	1.79^def^	11.25^a^	9.80^b^	11.03^ab^	3.50^c^	0.347	< 0.001
Total MUFA	18.15^f^	27.60^d^	37.69^a^	22.06^e^	20.04^ef^	14.57^g^	31.25^c^	34.36^b^	10.16^h^	18.96^f^	18.05^f^	20.73^ef^	0.690	< 0.001
Total C18:1 *c*	13.05^ef^	22.13^c^	30.69^a^	14.61^de^	16.23^d^	8.86^g^	14.72^de^	28.07^b^	5.79^h^	14.18^de^	13.83^de^	10.70^fg^	0.644	< 0.001
Total C18:1 *t*	3.78^ab^	4.10^ab^	5.04^a^	5.58^a^	2.06^b^	3.69^ab^	5.89^a^	3.65^ab^	1.95^b^	2.37^b^	2.13^b^	1.74^b^	0.557	< 0.001
Total PUFA	24.42^a^	15.29^de^	8.80^g^	19.96^bc^	17.42^cd^	11.20^fg^	9.21^fg^	22.25^ab^	10.87^fg^	8.71^g^	7.96^g^	13.28^ef^	0.918	< 0.001
Total CLA	0.12^ef^	0.17^def^	0.56^a^	0.52^ab^	0.24^bcde^	0.45^abc^	0.39^abcd^	0.70^a^	0.000^e^	0.000^e^	0.000^e^	0.000^e^	0.0690	< 0.001
Total n-3 FA	0.93^cd^	0.74^cd^	1.24^c^	0.59^d^	0.64^d^	1.12^cd^	0.80^cd^	1.02^cd^	2.10^b^	2.18^b^	1.78^b^	9.61^a^	0.123	< 0.001
Total n-6 FA	1.21^bc^	1.49^b^	0.74^ef^	1.02^cde^	1.00^cde^	0.88^def^	1.09^cd^	0.67^f^	1.20^bc^	1.28^bc^	1.46^b^	2.00^a^	0.073	< 0.001
Total n-3 FA/Total n-6 FA	0.77^cde^	0.50^e^	1.69^b^	0.56^e^	0.64^de^	1.28^bc^	0.73^cde^	1.51^b^	1.86^b^	1.83^b^	1.25^bcd^	4.81^a^	0.140	< 0.001
Total FA, g/kg DM	29.7^c^	72.7^abc^	111.3^ab^	61.3^abc^	46.3^bc^	25.7^c^	129.8^a^	43.7^bc^	15.8^c^	15.5^c^	10.9^c^	24.0^c^	15.62	< 0.001

Different superscripts within a row indicate significant differences among the tested meals (*P*-value < 0.05)

Abbreviations: *FA* Fatty acids, *ACD Acheta domesticus*, *ALD Alphitobius diaperinus*, *BL Blatta lateralis*, *GB Gryllus bimaculatus*, *GS Grylloides sigillatus*, *HI Hermetia illucens*, *MD Musca domestica*, *TM Tenebrio molitor*, *SBM* Soybean meal, *RPM* Rapeseed meal, *SFM* Sunflower meal, *FM* Fishmeal, *SEM* Standard error of the mean, *t Trans*, *c Cis*, *CLA* Conjugated linoleic acid, *SFA* Saturated fatty acids, *OCFA* Odd-chain fatty acids, *BCFA* Branched-chain fatty acids, *MUFA* Monounsaturated fatty acids, *PUFA* Polyunsaturated fatty acids, *DM* Dry matter

^1^Total BCFA: C13 *iso* + C13 *anteiso* + C14 *iso* + C15 *iso* + C15 *aiso* + C16 *iso* + C17 *iso* + C17 *aiso* + C18 *iso*

^2^Total C18:1 *cis*: (*c*9 + *c*10 + *t*15) + *c*11 + *c*12 + *c*13 + (*c*14 + *t*16) + *c*16

^3^Total C18:1 *trans*: *t*4 + *t*5 + (*t*6-8) + (*t*9-11) + *t*12 + (*t*13-14)

The proportion of total SFA in the rumen digesta ranged from 37.0 (TM) to 65.1 (HI) g/100 g FA, with no clear differences recorded when comparing the control (plant meals and FM) and insect meals. However, some meals showed different concentrations of some specific individual SFA. The HI rumen digesta contained the highest proportion of C12:0 (20.4 g/100 g FA), strongly differing from all the other meals (less than 1.5% of FA). The proportion of C14:0 in rumen digesta was the highest for FM (9.23 g/100 g FA), followed by HI (6.97 g/100 g FA), whereas the other meals showed values lower than 4.5 g/100 g FA. The proportion of C16:0 was the highest in GS (38.0 g/100 g FA) and the lowest in TM (23.2 g/100 g FA), the latter being not statistically different from SFM. The proportion of C18:0 was higher in ACD, BL, GS, and GB than in HI and TM (on average + 6.03 g/100 g FA), while the other meals showed intermediate values.

The proportion of total monounsaturated fatty acids (MUFA) was the lowest when SBM was incubated (9.8 g/100 g FA) and noticeably varied among the tested insect meals (up to 2.6-fold variations, from 14.9 to 39.3 g/100 g FA in HI and BL, respectively). The proportion of C18:1 *t*9-11 were the highest for GB, MD and BL (> 4.36 g/100 g FA) and was the lowest for FM (1.33 g/100 g FA). The proportion of C18:1 *c*9 (+ *c*10 + *t*15) was the highest for BL (31.3 g/100 g FA), followed by TM and ALD (> 22 g/100 g FA); HI and FM showed the lowest proportions of C18:1 *c*9 (on average 8 g/100 g FA).

The proportion of total PUFA was the highest for ACD (26.3 g/100 g FA; being not statistically different from TM) and the lowest for BL and RPM (9.4 and 10.2 g/100 g FA, respectively); as for total SFA, no clear differences were found when comparing the control and insect meals. However, when considering individual PUFA, the proportion of C18:2 n-6 for ACD rumen digesta (22.7 g/100 g FA) was the highest; and values > 10 g/100 g FA were detected also for ALD, GB, GS and TM. The highest proportions of C18:3 n-3 in the rumen digesta were found for plant meals (> 1.6 g/100 g FA). The proportions of C20:5 n-3 and C22:6 n-3 were the highest for FM.

The proportion of branched-chain fatty acids was higher when the plant meals (20.0 to 22.9 g/100 g FA) rather than the insect meals or FM (< 8 g/100 g FA) were incubated.

## Discussion

### Chemical composition and fatty acid profile of the meals

The chemical composition of the tested insect meals fell within the wide ranges reported in currently available reviews and research articles [42].

The full-fat insect meals used in this study showed a CP content comparable to that found in plant protein sources used in ruminant nutrition [6, 43]. The observed differences in the CP content among the insect meals could also be partially the consequence of variations in the nutritional quality of the rearing substrate, developmental stage of insects and industrial processing (e.g., drying) [44, 45]. Indeed, the tested insect meals were provided by different suppliers, the insects being raised and further processed under different conditions. Even though, the CP content of our samples were in line with those published in literature on the same species [42, 46]. When comparing the CP values of insect meals with published literature, the N:P conversion factor used for CP calculation must be considered [9]. When using the conventional N:P conversion factor of 6.25, it is assumed that the matrix under analysis contains 160 g N/kg CP, and that all N is of protein origin. However, in insects, part of the N is embedded in chitin; this N is also extracted when analysing proteins with the classical Kjeldahl methodology, leading to an overestimation of the real CP content [29]. For instance, when comparing the CP values of the ACD and GB meals used in our trial with the values obtained for other ACD and GB meals by Ahmed et al. [8], our values seem lower, but result similar when using the same N:P conversion factors [30] (49.9 and 45.2 g/100 g DM for ACD and GB, respectively).

Common as well as alternative protein feeds used in ruminant nutrition are naturally characterized by low amounts of fat or they are by-products of lipid extraction [23]. The tested insect meals were instead rich in EE (> 200 g/kg DM). Such values were consistent with data reported for full-fat meals obtained from the same insect species by other authors (e.g., [42, 46, 47]).

The FA profiles differed between the meals and were consistent with published data [43, 48]. Many insects can endogenously synthesize medium- and long-chain SFA, such as C14:0, C16:0 and C18:0 [49]. The general high proportion of SFA in insect meals could pose concerns due to the expected negative impacts on the nutraceutical quality of ruminant-derived food products [50], as already observed in food products from monogastric animals when including insect meals other than TM in their diet [51, 52]. The presence of the enzyme Δ9-desaturase (already described in Dipteran species such as *Drosophila melanogaster* and *M. domestica*, and recently hypothesised also for *H. illucens*) would enable the conversion of these SFA into the respective *cis*9-MUFA, justifying the high levels of C18:1 *c*9 found in all the insect meals in the current study [53]. The high proportion of SFA, and especially C12:0, observed in HI was also observed by Jayanegara et al. [18] and Ahmed et al. [8]. This peculiar characteristic is most probably the consequence of the subtropical origin of this species. In fact, it has been hypothesised that the high melting point of SFA could enable HI to prevent lipid oxidation and to survive at the typical high temperatures of subtropical areas [54]. The main FA found in GB, TM, MD and ACD meals agreed with the findings of other authors [18, 55, 56]. Consistently with the results obtained in our study, Oonincx et al. [57] showed that the main individual FA in full-fat ALD larvae were C16:0, C18:1 n-9 and C18:2 n-6, while only low levels of C18:3 n-3 are typically found in this species. The high C18:2 n-6 content in ACD is most probably the consequence of the presence of a Δ12-desaturase in this species [55]. Data about the FA profile of BL quite differed from the findings of Kulma et al. [46], who reported higher MUFA and lower PUFA proportions (42.2 and 9.6 g/100 g total FA, respectively) for BL subadults when compared to our results. Such differences are most probably imputable to the insect rearing substrate: Kulma et al. [46] fed BL with dog granules and old bread ad libitum, with an additional daily provision of slices of fresh vegetables and fruits, which differed from the rearing substrate (mainly a mixture of vegetables and fruits, added with old bread, breadsticks and brioches) used to raise BL in our trial.

### In vitro rumen fermentation parameters

In our study, the incubation of insect meals resulted in lower gas and VFA production than did that of the plant meals. Previous studies [19, 58] also observed a reduction in gas and VFA production with different insect meals when compared to SBM. These reductions could be, first, explained by the chemical composition of the insect meals, as they contained high CP and EE contents. Protein and fat contribute little to gas and VFA production [59] and could even alter the ruminal fermentations. Indeed, negative effect of CP on fermentation is attributed to the buffer capacity of proteins, whereas a high fat content may lead to inhibition of rumen microbes and decreases in carbohydrates digestibility [60]. The high final pH observed with insect meals is typical of high CP and EE contents and low gas production. Because of the low gas and VFA production, poor IVOMD values were obtained in our study for the insect meals. Jayanegara et al. [58] suggested that the chitin content could also explain the low gas and VFA production with insect meals, as chitin would not be degraded by ruminal microorganism. When using insect meals in animal nutrition, it has been shown that chitin can depress diet digestibility, as some animal species do not contain chitinase activity [14]. Tabata et al. [61] showed that the levels of acidic chitinase mRNA in the bovine stomach tissues are very low; however, the same authors hypothesised that chitinases may be provided by the bacteria populating the gastrointestinal tract of ruminants, thus playing a possible role in chitin digestion. Indeed, chitinase activity has been described in some rumen bacteria and protozoa [62] and Fadel El-Seed et al. [63], using the in situ technique, observed that chitin was degraded in the rumen of sheep but at a very low rate of degradation. Indeed, the fermentation parameters were higher for BL in comparison to the other insect meals despite BL had the highest chitin content and a very high EE content. This suggests that other factors could explain the low gas and VFA production and IVOMD with insect meals. The BL meal was the only produced from subadult insects, and the developmental stage of the insects is known to affect their chemical composition and fermentation parameters. Indeed, Jayanegara et al. [58] observed differences in gas (– 34 mL/g DM after 24 h incubation) and VFA production (– 66.5 mmol/L) and in IVOMD (– 0.07 g/g) between HI meals at 1 week or 2 weeks larval stage. The exoskeleton of BL might be more degradable than those of the other insect larvae used in this study; such a hypothesis needs further investigations. The low unsaturated FA (UFA) concentration in BL meal compared to the other tested insect meals could also have less inhibited the rumen fermentations [64], being BL meal characterised by high SFA, and particularly rich in C16:0. However, HI was also characterised by high SFA content (mainly C12:0) but resulted in low gas and VFA production. Hristov et al. [65] observed a depressive effect in VFA production with the inclusion of C12:0 in the rumen. This suggest that chitin, CP and EE contents, as well the lipids FA composition may interact in determining the ruminal degradation process of different insect meals.

#### Methane production

Compared to plant meals, methane production was reduced with insect meals due to a reduction in gas production. However, a decrease in the proportion of methane in total gas for GB, HI, MD and ACD meals was observed as well, suggesting a specific effect on methanogenesis. Ahmed et al. [8] reported also a potential direct inhibition of methanogenesis by GB. The ACD, GB and MD meals had a high content of UFA, which are known to contribute to methane reduction through a toxic effect on microbial populations [64]. The HI meal contained mainly C12:0 and C14:0, and some oils rich in C12:0 and C14:0 have also been suggested to reduce ruminal methane production [66].

Jayanagera et al. [19, 58] suggested that chitin and its derivative chitosan may play a role in methane reduction, but this hypothesis seems not confirmed by our data. The effects of chitin and chitosan on rumen methanogenesis have been limitedly investigated to date and recent publications [18, 67] have shown a lack of agreement on the effects of chitin and chitosan on methane mitigation potential.

#### Ammonia production and protein value of insect meals

High ammonia production was related to higher CP content except for FM. However, when ammonia was expressed as proportion of total N, insect meals had lower ammonia production than plant meals (except BL), meaning that protein from insects would be less degradable. Toral et al. [9] also reported a lower ruminal protein degradation for four different insect meals (TM, *Zophobas morio*, ALD and ACD) when compared to SBM. Fishmeal, which is known to be mainly a source of undegradable protein [68], had one of the lowest ammonia productions. Low protein degradation with insect meals could be explained by an important proportion of protein associated to chitin and chemically linked to the exoskeleton of insects [69]. The BL meal had higher ammonia production than the other insect meals, meaning that protein from BL would be more degraded in the rumen, confirming the high ruminal degradation deduced through gas and VFA production.

A low protein ruminal degradation enables to increase the intestinal protein flow, which can be favorable for animal production provided that intestinal digestibility is high. Our results suggested that insect meals, except BL, would be sources of rumen undegradable proteins (RUP). It is likely that part of the RUP from insect meals would be non-digestible proteins associated to chitin, especially considering that Tabata et al. [61] measured a low level of acidic chitinase mRNA in bovine abomasum compared to other animal species. Moreover, a high fat content can also reduce protein intestinal digestibility. Toral et al. [9] measured high and similar in vitro intestinal digestibility of the non-degraded ruminal proteins from TM, ACD and ALD (between 0.64 g/g and 0.78 g/g) as SBM (0.68 g/g), whereas Ioselevich et al. [70] measured a lower protein intestinal digestibility (0.53 g/g) for *Bombyx mori* pupae. The lack of available data on ruminal degradation and intestinal digestibility of proteins from insect meals and the inconsistent available results underline the need of further protein evaluation studies to better assess this process.

### Fatty acid profile of rumen digesta

Thanks to their high EE content, full-fat insect meals may represent a potential source of lipids, alternative to plant oils, to increase the energy density of diets and/or to improve the FA profile of animal-derived food products [71, 72]. This last point could be interesting, as most of the insect species we studied resulted rich in UFA. The ACD, TM, GB, GS, and ALD kept indeed higher concentration of C18:2 n-6 and other FA with potential health-promoting effects (e.g., C18:1 *t*11 or CLA *c*9*t*11; [73]) than plant meals as unaltered after ruminal biohydrogenation. The low biohydrogenation of PUFA from insect meals can be explained by a possible inhibitory effect of the high amount of fat (the total FA content in the tested insect meals was on average 10 times higher than that of the tested plant meals) on ruminal microbial populations [73]. This can also be the explanation for the much higher total branched-chain fatty acids content found in the ruminal digesta when the plant meals rather than the insect meals were incubated [74]. However, the EE content alone would not be sufficient to justify the low ruminal biohydrogenation of PUFA from insect meals, as for BL and TM the C18:2 n-6 biohydrogenation rate was high even if such meals showed high EE content. Besides the EE content, the FA composition and unsaturation can also explain the protection of PUFA from in vitro ruminal biohydrogenation. The protective effect was important only for insect meals rich in PUFA (ACD, TM, GS), whereas when the EE had SFA or medium-chain MUFA as main constituents (as for BL, HI and MD), the degree of PUFA biohydrogenation was similar to that of the plant meals. The inhibitory effect of dietary PUFA on ruminal microbial population responsible for their biohydrogenation is well known and documented in literature for lipid supplements of plant origin [75, 76]. Our results are also in agreement with the low biohydrogenation rate of insect oil PUFA recently observed by Hervás et al. [22].

In terms of the potential beneficial effects on animal product quality, the HI meal seems to be the less attracting, as its rumen digesta was rich in SFA, and in particular in C12:0 and C14:0, which are transferred to milk and meat without being further modified [65, 76]. When dealing with full-fat insect meals, a higher total SFA concentration should be expected in the rumen digesta when compared to the same for plant meals, due to their noticeable differences in terms of total lipid content. This aspect should also be considered when hypothesizing the use of full-fat insect meals to improve the lipid quality of ruminant-derived food products.

## Conclusion

In conclusion, to the best of our knowledge, this is the first article presenting data on the in vitro ruminal digestibility of BL, GS and MD meals. Whatever the insect species, the full-fat insect meals appear to be potentially an interesting feed source, alternative to the most common ones of plant origin. The in vitro organic matter disappearannce of the tested insect meals was lower than that of plant meals, probably because of their high fat content and its FA composition. Chitin may also have played a role. The observed lower ammonia content of rumen fluid for the insect meals when compared to that of the plant meals could be an advantage, provided that intestinal digestibility is high. Further studies are required to better explore the intestinal digestibility of insect meals and the technological solutions to increase their overall digestibility. The FA profile of rumen digesta of ACD, ALD, GB, GS and TM, being rich in n-6 PUFA, could be interesting to potentially improve the quality of derived ruminant products. This research suggests that these innovative feedstuffs could have a potential interest as substitutes for more conventional protein and fat sources in ruminant diets.

## Supplementary Information

**Additional file 1.** Coelution and separation of C18:1 *t*9 to *t*11 (g/100 g FA) of ruminal digesta after 24 h incubation of the insect and control meals.

## Data Availability

The datasets analysed in the current study are available from the corresponding author on request.

## Abbreviations

ACD: *Acheta domesticus*
ADF: Acid detergent fibre
ADIN: Acid detergent insoluble nitrogen
ADL: Acid detergent lignin
ALD: *Alphitobius diaperinus*
BL: *Blatta lateralis*
CP: Crude protein
DM: Dry matter
EE: Ether extract
FA: Fatty acid
FAME: Fatty acid methyl ester
FM: Fishmeal
GB: *Gryllus bimaculatus*
GS: *Grylloides sigillatus*
HI: *Hermetia illucens*
IVOMD: In vitro organic matter disappearance
MD: *Musca domestica*
MUFA: Monounsaturated fatty acids
NDF: Neutral detergent fibre
NDIN: Neutral detergent insoluble nitrogen
OCFA: odd-chain fatty acids
PUFA: Polyunsaturated fatty acids
RPM: Rapeseed meal
RUP: Rumen undegradable protein
SBM: Soybean meal
SFA: Saturated fatty acids
SFM: Sunflower meal
TM: *Tenebrio molitor*
UFA: Unsaturated fatty acids
VFA: Volatile fatty acids
