# Supplementary material for: Full-fat insect meals in ruminant nutrition: in vitro rumen fermentation characteristics and lipid biohydrogenation
Source: J Anim Sci Biotechnol. 2022 Dec 20;13:138. doi: 10.1186/s40104-022-00792-2 (PMC9764709; doi:10.1186/s40104-022-00792-2)
Supplement: Supplementary file 1 — Additional file 1. Coelution and separation of C18:1 t9 to t11 (g/100 g FA) of ruminal digesta after 24 h incubation of the insect and control meals. [file 40104_2022_792_MOESM1_ESM.docx]

## Additional file 1. Coelution and separation of C18:1 *t*9 to *t*11 (g/100 g FA) of ruminal digesta after 24 h incubation of the insect and control meals.

|  | C18:1 *t*9 | | C18:1 *t*10 | | C18:1 *t*11 | | C18:1 *t*9+*t*10 | | C18:1 *t*10+*t*11 | |
| --- | --- | --- | --- | --- | --- | --- | --- | --- | --- | --- |
|  | mean | S.E. | mean | S.E. | mean | S.E. | mean | S.E. | mean | S.E. |
| ACD | 0.48 | 0.014 |  |  |  |  |  |  | 2.65 | 0.503 |
| ALD |  |  |  |  | 1.70 | 0.49 | 1.87 | 0.16 |  |  |
| BL | 0.37 | 0.025 | *1.02* | *-* | *3.36* | *-* |  |  | 3.63 | 0.404 |
| GB | 0.56 | 0.145 | 1.24 | 0.336 | 2.59 | 0.658 | *2.188* | *-* |  |  |
| GS |  |  |  |  | 0.79 | 0.012 | 1.02 | 0.020 |  |  |
| HI | 0.21 | 0.014 | 0.47 | 0.044 | 2.46 | 0.158 |  |  |  |  |
| MD | 0.55 | 0.094 | 0.89 | 0.231 | 2.97 | 0.433 |  |  |  |  |
| TM | 0.14 | 0.018 |  |  |  |  |  |  | 3.15 | 0.700 |
| SBM | 0.13 | 0.014 | 0.20 | 0.024 | 1.40 | 0.193 |  |  |  |  |
| RPM | 0.21 | 0.014 | 0.24 | 0.039 | 1.49 | 0.081 |  |  |  |  |
| SFM | 0.15 | 0.016 | 0.24 | 0.018 | 1.42 | 0.089 |  |  |  |  |
| FM | 0.24 | 0.037 | 0.26 | 0.033 | 0.82 | 0.075 |  |  |  |  |

## Abbreviations. FA: fatty acids; ACD: *Acheta domesticus*; ALD: *Alphitobius diaperinus*; BL: *Blatta lateralis*; GB: *Gryllus bimaculatus*; GS: *Grylloides sigillatus*; HI: *Hermetia illucens*; MD: *Musca domestica*; TM: *Tenebrio molitor*; SBM: soybean meal; RPM: rapeseed meal; SFM: sunflower meal; FM: fish meal; *t*: *trans*; S.E.: standard error.

## Values in italic are issued from only one sample out of 4.
